# On the Possibility of Reproducing Utsu’s Law for Earthquakes with a Spring-Block SOC Model

**DOI:** 10.3390/e25050816

**Published:** 2023-05-18

**Authors:** Alfredo Salinas-Martínez, Jennifer Perez-Oregon, Ana María Aguilar-Molina, Alejandro Muñoz-Diosdado, Fernando Angulo-Brown

**Affiliations:** 1 Departamento de Física, ESFM, Instituto Politécnico Nacional, Mexico City 07738, Mexico; 2 Departamento de Ciencias e Ingeniería, Instituto Tecnológico y de Estudios Superiores de Monterrey, Ciudad López Mateos 52926, Edo. de México, Mexico; 3 Departamento de Ciencias Básicas, UPIBI, Instituto Politécnico Nacional, Mexico City 07340, Mexico

**Keywords:** self-organized criticality, spring-block, seismicity, Utsu law, aftershocks

## Abstract

The Olami, Feder and Christensen (OFC) spring-block model has proven to be a powerful tool for analyzing and comparing synthetic and real earthquakes. This work proposes the possible reproduction of Utsu’s law for earthquakes in the OFC model. Based on our previous works, several simulations characterizing real seismic regions were performed. We located the maximum earthquake in these regions and applied Utsu’s formulae to identify a possible aftershock area and made comparisons between synthetic and real earthquakes. The research compares several equations to calculate the aftershock area and proposes a new one with the available data. Subsequently, the team performed new simulations and chose a mainshock to analyze the behavior of the surrounding events, so as to identify whether they could be catalogued as aftershocks and relate them to the aftershock area previously determined using the formula proposed. Additionally, the spatial location of those events was considered in order to classify them as aftershocks. Finally, we plot the epicenters of the mainshock, and the possible aftershocks comprised in the calculated area resembling the original work of Utsu. Having analyzed the results, it is likely to say that Utsu’s law is reproducible using a spring-block model with a self-organized criticality (SOC) model.

## 1. Introduction

In the present article we propose a method to estimate the aftershock area given by the Utsu law [1,2] by using the spring-block model suggested by Olami, Feder and Christensen (OFC) [3] based on a bidimensional spring-block array capable of reaching a self-organized criticality (SOC) state. As is well known, the SOC concept was developed by Bak et al. in 1987 [4,5,6,7] and since then this idea has been useful for many applications in several disciplines [8]. The OFC model works as a cellular automaton with a few rules for its evolution (see Section 2.1). The first property known to be reproduced by this model was the Gutenberg—Richter law [9] $log\dot{N}\left(M\geq M_{0}\right)=a-bM$, which relates the magnitude of an earthquake to the number of earthquakes of a given magnitude M, where a is a constant and b is the scaling parameter between large and small events and varies depending on the subduction zone. For several years it was believed that this model did not reproduce aftershocks. However, in 2002 Hergarten and Neugebauer (HN) [10] proposed a manner to obtain aftershocks with the original OFC model.

Aftershocks and their decay have been studied for a long time. Omori proposed in 1894 that they follow a hyperbolic decay with time, following $n=\frac{k}{\left(c+t\right)}$, which is known as the Omori law [11], where n is the number of aftershocks; k and c are constants that vary between earthquake sequences; and t is the time elapsed since the mainshock. Later, this equation was modified by Utsu in 1961 and took the general form of $n=\frac{k}{{\left(c+t\right)}^{p}}$ which is known as the Omori-Utsu law [1], where p is a constant that determines the decay rate and can take values between 0.6 and 1.1 [12] if the aftershock sequence is modeled by means of a multifractal scaling theory. However, Davidsen et al. [13], by using a generalized Omori-Utsu law, found that the exponent p is a constant >1 providing convincing evidence that the characteristic time c is not constant but a genuine function of $m_{c}$, with $m_{c}$ being at a lower magnitude cut off for the aftershocks considered. Some authors have tried to give a generalized form of this law in the form of a differential equation and introduced the idea of a “cool down” process after the mainshock [14,15]. In the end, the goal has been to characterize the behavior of aftershocks so the general activity can be predicted after a big event and therefore, asses the risk in a given zone [16]. In addition, there seems to be a relationship between the parameter b of Gutenberg—Richter and the parameter p of Omori which governs the speed of the aftershock’s decay [17,18,19,20]. So far, we know that the Gutenberg—Richter law is reproducible in the OFC model, so we aimed to see if some sort of Omori-like behavior could be also obtained.

Our group of researchers has studied for a long time the OFC model, trying to reproduce real-seismicity properties reported by seismologists. For our part, we have published several articles in which we found some similarities between the OFC model and real seismicity [3,21,22,23,24,25,26,27,28]. We can outline the ability to emulate real-life seismic subduction zones by including the age of the lithospheric plate and the rate of convergence, both transformed into their synthetic counterparts in the spring-block model [21]; we were also able to reproduce the so-called Ruff—Kanamori diagram [21,23,29] that relates the age of the subduction plate with the convergence rate of the plates and the maximum characteristic earthquake for a given zone. In a different paper, we suggested that the Gutenberg—Richter parameters a and b are positively correlated [24]. We have also published a variation of the OFC model, including non-homogenous cases for different elastic ratios [25,27] and getting the asperity concept into the spring-block model. In the same article, we present an extensive review of our previous results.

In terms of the current study, it aims to replicate Utsu’s law for earthquakes within the OFC model. Additionally, in the spring-block model, there is potential to identify certain events as aftershocks by considering their spatial location.

In the work that our research group has performed, we conducted simulations to study real seismic regions, analyzed the maximum earthquake and identified possible aftershock areas using Utsu’s formulae. We proposed a new equation to calculate the aftershock area and analyzed the behavior of the surrounding events to determine whether they could be classified as aftershocks. By considering the spatial location of these events, we plotted the epicenters of the mainshock and the possible aftershocks within the calculated area, concluding that Utsu’s law is reproducible using a spring-block SOC model. 

As for the remaining work, evaluating the suitability of aftershock candidates according to Omori’s law is required. Moreover, exploring the inclusion of the spatial location in identifying potential aftershocks also needs a deeper examination, as we will discuss further.

This article is organized as follows: in Section 2, we present the OFC model and Utsu’s equations. In Section 3, we present the results of the simulations performed to characterize the different seismic regions and the relationship between the different equations to calculate the aftershock area. In Section 4, we discuss the results and analyze them to identify possible aftershocks and the application of the equations for the aftershock area and, finally, in Section 5, we present our conclusions.

## 2. Methodology

### 2.1. The Olami-Feder-Christensen Model

**Figure 1:**
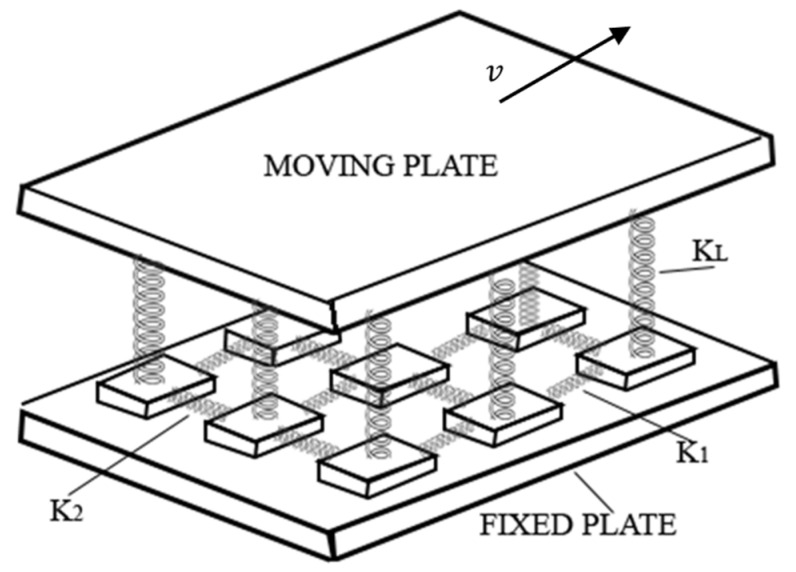
Schematics for the spring-block model.

The OFC model is characterized as being a non-conservative continuous model that displays self-organized criticality [3]. It is made by a system of blocks interconnected by Hooke springs. Each block is connected to four nearest neighbors. In addition, each of these blocks is connected to a moving plate by another set of Hooke springs and connected to a fixed rigid plate, as shown in Figure 1. The block’s displacement is given by the relative movement between the two plates; when the force in one of the blocks overcomes a certain threshold $F_{th}$ (the maximum static friction), then the block slips. The moving block then returns to the zero-force position after transferring its energy to the four nearest neighbors which can result in further slips that can provoke a chain reaction. 

The spring-block model is a two-dimensional dynamic system composed of interconnected blocks and springs. The blocks are connected to a rigid plate that moves at a constant speed, and the relative motion of the blocks causes them to move as well. Each block has a maximum of four neighbors and will slip if the force acting on it exceeds a certain threshold value. The model can be described using cellular automata, where each block has a force and displacement from its relaxed position on the lattice. The movement of a slipping block can trigger a chain reaction that results in more block movements.

In order to generate the synthetic earthquakes, the spring-block model is mapped to a continuous, non-conservative cellular automaton that follows the next computational evolution rules, as described below.

1. The lattice sites are initialized with random values between zero and a force threshold $F_{th}.$
2. The block with the largest force, $F_{max},$ is located, and the difference between $F_{th}$ and $F_{max}$ is added to all sites. This causes a global perturbation.
3. For all sites where $F_{i,j}\geq F_{th}$, the force is redistributed among the neighbors of $F_{i,j}$ according to the following rule: $F_{n,n}$, the forces of the neighboring blocks of the relaxed cell $F_{i,j}$ are increased by $\gamma F_{i,j}$, and $F_{i,j}$ is reduced to zero.
4. Step 3 is repeated until the earthquake has fully evolved.
5. Once a state of rest is reached, the process returns to step 2, and synthetic earthquakes are observed until the desired number of events is reached.

The computational rules described before are deduced from the dynamics of the OFC model, which are the following: Consider a network of size L×L, where each block will have a force of $F_{i,j}$ applied to it, with i and j being integers between 1 and L. The displacement of each block from its relaxed position on the lattice will be denoted as $x_{i,j}$ [3].

The total force exerted on a specific block (i,j) by the springs can be expressed as:

(1) $$F_{i,j}=K_{1}\left[{2x}_{i,j}-x_{i-1,j}-x_{i+1,j}\right]+K_{2}\left[{2x}_{i,j}-x_{i,j-1}{-x}_{i,j+1}\right]+K_{L}x_{i,j}$$

where $K_{1}$, $K_{2}$, $K_{L}$ are the elastic constants.

Once a local slip at the position (i,j) has happened, the force will redistribute in the following way:

(2) $$F_{i\pm 1,j}\to F_{i\pm 1,j}+{\delta F}_{i\pm 1,j}$$

(3) $$F_{i,j\pm 1}\to F_{i,j\pm 1}+{\delta F}_{i,j\pm 1}$$

(4) $$F_{i,j}\to 0$$

where the force increase in the nearest neighbors is given by:

(5) $$\delta F_{i\pm 1,j}=\frac{K_{1}}{{2K}_{1}+{2K}_{2}+K_{L}}F_{i,j}=\gamma_{1}F_{i,j}$$

(6) $$\delta F_{i,j\pm 1}=\frac{K_{2}}{{2K}_{1}+{2K}_{2}+K_{L}}F_{i,j}=\gamma_{2}F_{i,j}$$

where $\gamma_{1}$ and $\gamma_{2}$ are the elastic ratios.

If $K_{L}$ is greater than 0, the force redistribution is not conserved, which is expected during actual earthquakes. This results in a redefinition of forces in the nearest-neighbor blocks, which may cause further slipping and trigger a chain reaction or avalanche. For the isotropic case where $\gamma_{1}=\gamma_{2}=\gamma$, the OFC model can reproduce the Gutenberg—Richter power law with an exponent similar to the actual one for γ equal to 0.2. Elastic ratios, defined in Equations (5) and (6) can take values between 0 and 0.25. However, the values of the parameter b in the Gutenberg—Richter law that are similar to those observed in real seismicity are only obtained for γ around 0.2.

The magnitude M of a synthetic earthquake will be defined as a function of the number n of relaxed blocks, where $M=log_{x}(n)$, and x is the base of the logarithm, in this way the size of the synthetic earthquake is determined by n.

For a more detailed description of the equations and to deepen the choice of elastic parameters, we recommend reading reference [9] where we present an extensive review on this topic.

Based on the work of Perez Oregon et al. [21,22,23], in which they simulated several seismic regions considering the age of the subduction plate and the convergence velocity to reproduce the maximum magnitude earthquake for each region and also using the OFC spring-block model, 29 simulations were performed, same as in [21], using lattices of 100 × 100 blocks and one million iterations. The synthetic age of the subduction zone was obtained considering the interval from 0 to 160 Myr proposed by Ruff and Kanamori [29] and defining $e_{n}$ as the normalized age of the tectonic plates. If we set the value to 1 for the maximum value of the tectonic age (160 Myr) we propose a relation of γ and the tectonic age using [21]:

(7) $$\gamma =0.25(1-e_{n})$$

It can be seen that for an old plate γ takes lower values that transform to low-magnitude earthquakes, while for younger ones γ is close to 0.25, which generates high-magnitude earthquakes. For each of the subduction regions considered, the value of γ was calculated. In addition, we included the convergence rate for these plates ranging from 1 to 11.1 cm/year as reported in [23] but converted to the synthetic counterpart ranging from 0 to 3. This synthetic velocity was implemented in the spring-block model by adding it to the difference between $F_{th}-F_{max}$ in the first iteration as a global perturbation to obtain the maximum event per simulation.

The magnitude of the synthetic earthquake was determined using the ${log}_{3}$ of the size of the earthquake; this size is referred to as the number of relaxed blocks during the simulation. The value of ${log}_{3}$ was chosen arbitrarily, and it gives the closest value to real magnitudes [23,25]. We want to emphasize that this log value may change if the size of the lattice changes or the number of iterations. Therefore, a new value must be chosen to match the values of real earthquakes. For example, in a 100 × 100 lattice in the case that for a single event all the blocks were relaxed, we would have $M=\log_{3}(10000)=8.3$; while for a 500 × 500 lattice, the same scenario would be $M=\log_{3}(250000)=11.3$, which is by far greater than any earthquake reported. In this case, there is a great level of correspondence between the synthetic and the real values of the earthquakes’ magnitudes for each region, as reported in [23,25]. 

### 2.2. Utsu’s Law

In its original publication [2], Utsu proposes that every earthquake taking place in a one-month period after the main event and where the magnitude is less than the main event’s magnitude should be considered as an aftershock and not as an isolated event. He also proposes a region in which the epicenters of these aftershocks should be located. This region is called the aftershock area. Utsu [2] defines the aftershock area as the physical area in which the aftershocks are embedded.

The first relationship between the earthquake’s magnitude and the aftershock area was proposed in 1955 by Utsu and Seki [30] as shown in Equation (8):

(8) logA=1.02M−4.01

where *A* is the aftershock area in km^2^, and *M* is the earthquake magnitude and log is a base 10 logarithm.

However, this relation is not unique; for different magnitudes there are different equations which provide a better adjustment. In general, the equations take the general form of logA=∝M−β, varying the constants ∝ and β according to the case.

For magnitudes M<7, he proposes:

(9) logA=M−3.7.

This equation was originally used as a correction of Equation (8) in a zone where the aftershocks exhibit a large scatter [2].

For inland earthquakes, he found that the aftershock area is smaller to offshore earthquakes, and that they fit closer to the following equation:

(10) logA=M−4.1.

This is valid for earthquakes with magnitudes 5.5≤M≤7.5.

Goto [31] proposes an equation for the minimum aftershock area for M>6.5:

(11) logA=1.74M−9.92,

valid for regions where the seismic activity is low. However, Utsu mentions that the aftershock area given by the latter equation is too small, therefore, he proposes a better adjustment using:

(12) logA=M−4.4,

which is valid for 5.5≤M≤8.

On the other hand, Bath and Buda [32] propose the following relation after analyzing six earthquakes:

(13) logA=1.21M−5.05

In addition, Purcaru [33] proposes a relation for 5≤M≤8.5 using the data of fifteen earthquakes:

(14) log⁡A=1.08M−4.17

### 2.3. Aftershocks in the OFC Model

Some researchers [10,34] have pointed out that one of the biggest limitations of the OFC model is the lack of foreshocks or aftershocks, which, in this case, may be difficult to prove that Utsu’s law is present in the OFC model. Some authors have tried to solve this problem. Before, we can mention the work of Hainzl et al. [35] who introduced an additional set of blocks representing the viscous asthenosphere; Pelletier [34], based on Hainzl’s work [35], included heterogeneities into his model. In both cases, they modified the original OFC model and made it more complicated by introducing these new variables. Hergarten and Neugebauer [10] did a statistical analysis of the time series in the OFC model and proposed a method to classify events after the mainshock as aftershocks. They proposed to use the time (number of events) between what they consider successive mainshocks of the same or larger magnitude as the recurrence time. Then, they set a window to limit where to look for aftershocks. After this, as long as the following events are not bigger than the mainshock and are located within this window, they are considered aftershocks.

## 3. Results

### 3.1. Utsu’s Law Simulations

**Figure 2:**
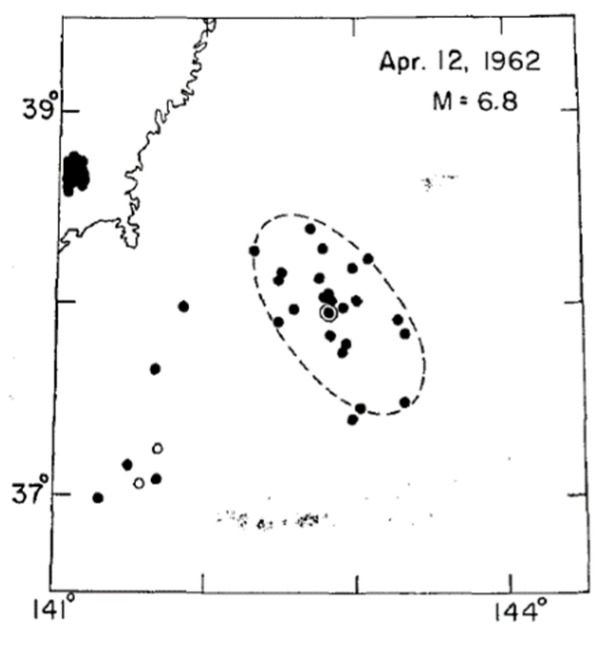
The ellipse in the dashed line represents the aftershock area proposed by Utsu. All the events with epicenters inside this ellipse are considered aftershocks of the main event that is marked as a double circle. Image taken from [1].

**Figure 3:**
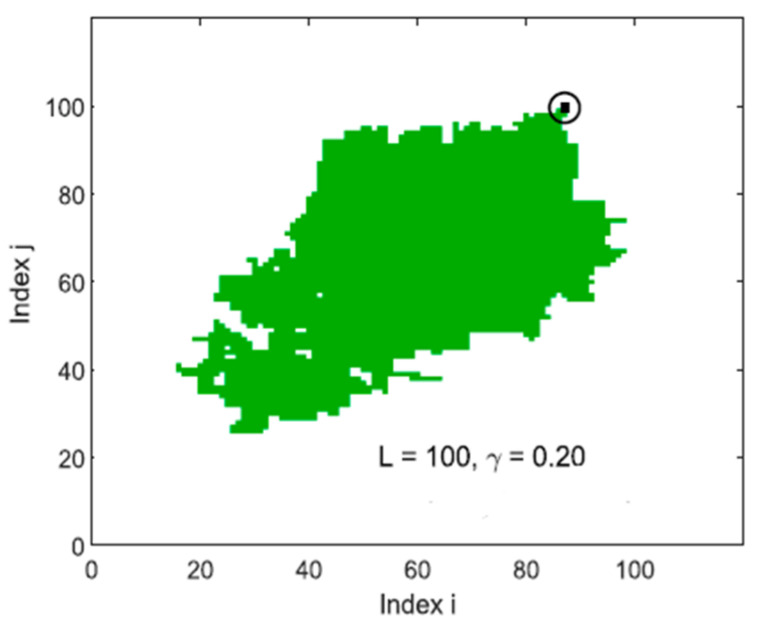
Relaxed area in a spring-block simulation. The earthquake’s epicenter is shown as a black dot in the black circle. L is the size of the lattice, γ is the elastic parameter or conservation level [24].

In Figure 2, we show one of the cases in the original work of Utsu where the main shock is illustrated as a double circle while the aftershock area is the dashed line ellipse, all the events that fulfill the requirements of time and magnitude are considered aftershocks [2]. The relative position of the main shock with respect to the aftershocks in the twenty-five cases reported by Utsu in [1] are located in some cases at the edges of the respective area, but in many of them they are located practically in the middle of the area. For the spring-block simulation, we take into consideration that the relaxed elements in our simulations can be identified graphically, and these elements generate a “relaxed area” equivalent to the size of the synthetic earthquake (Figure 3); we propose the equivalence between the synthetic earthquake’s size and Utsu’s aftershock area. When a synthetic earthquake occurs, the relaxed area has many blocks that are near the threshold, therefore, the probability that the following earthquakes occur in that area is high, which is in consonance with real seismicity. Based on this, we take the relaxed area as equivalent to the aftershock area in the Utsu construction.

In order to compare the real magnitude (the maximum magnitude reported in a given seismic region) and the real aftershock area versus the synthetic magnitude and the synthetic aftershock area, we treated real earthquakes’ magnitudes as synthetic ones in a spring-block simulation so we can expect a certain size (synthetic) for those events (where this size is the number of relaxed elements in a simulation). Since we already have emulated 29 subduction seismic zones [21,25], it was easy to apply the following transformation for going from real seismicity to synthetic seismicity:

(15) $$\mathrm{Expected}\;\mathrm{Size}\equiv \hat{S}=3^{M}$$

Afterwards, we calculated the aftershock area using Equations (8)–(14) and (16) (see below) as shown in Table 1. Table 1 lists the maximum real earthquake’s magnitude reported for each one of the twenty-nine subduction seismic regions that were used in [21]. We highlighted the equation that gives the closest value to the expected size calculated with Equation (16) so the reader can identify the best fit for each case. This comparison was made only with Equations (8)–(14). In addition, we obtained a new equation using linear regression for this data set. The resultant equation was:

(16) log⁡A=0.477M

It is worth mentioning that our proposed Equation (16) is the one closest to the expected size in comparison to the rest of Equations (8)–(14) and it is the reason the comparison mentioned earlier did not include Equation (16). As the magnitude increases, the difference between the expected size and the fit given by Equations (8)–(14) also increases dramatically. On the other hand, that difference remains minimal with Equation (16).

In Table 1, we present the results for the maximum earthquake’s magnitude reported (real seismicity), while in Table 2 are the results for the spring-block simulations of each seismic region (synthetic seismicity). For Table 2, we present the maximum synthetic size which was obtained with the spring-block simulation and then the equivalent magnitude was computed with Equation (15) and solved for M. The reader might notice that the same magnitude has associated different values of the synthetic size; this is the result of rounding Equation (15) to one decimal position. In both cases, as previously stated, the earthquake’s size is equivalent to the aftershock area, so this value should be approximated by any of the Equations (8)–(14). We highlighted the computed aftershock area that is the closest to the earthquake’s size by comparing Equations (8)–(14) so the reader can identify the best fit for each magnitude. 

In Table 1, according to our data model, a particular equation fits more accurately to a certain magnitude range. For magnitudes M<7.6, the closest result is given by Equation (8); while in the range of 7.6≤M≤8, the best adjustment is given by Equation (10) and for magnitudes over 8, the best adjustment is given by Equation (12). However, it is important to mention that, in all ranges in our proposal, Equation (16) is almost equal to the expected size.

In Table 2, since there are slight variations in the synthetic earthquakes (for example, we have the lowest magnitude value of 6.8 in comparison with 7.5 in Table 1) the equations also have variations over the range they fit the best. We can see that for M=6.8, Equation (13) gives the best adjustment; on the other hand, for M=7.2 we have two adjustments (again, given by the rounding of Equation (15)), the first is given by Equation (9), and the second one by Equation (8). The behavior for M≥7.6 is identical to the one in Table 1. For the synthetic catalog, we found that in our proposal, Equation (16) gives the most accurate value for the synthetic size.

**Figure 4:**
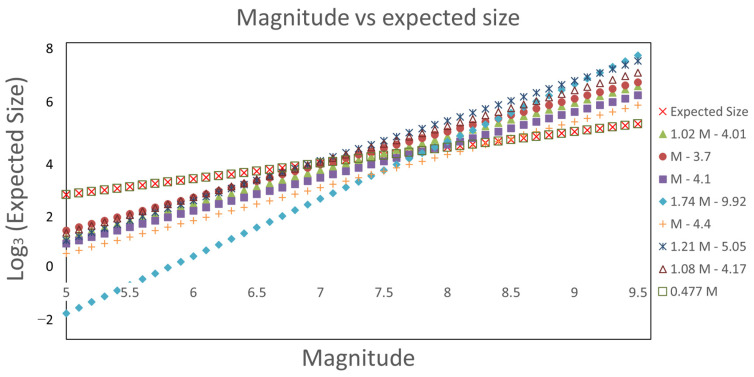
Magnitude vs. ${log}_{3}$ (expected size) given by Equations (8)–(14).

In Figure 4, we show a projection of Equations (8)–(14) and (16) for magnitudes 5≤M≤9.5 vs. the ${log}_{3}$ of the synthetic size in the spring-block model. On the one hand, it can be noticed that in the range of 6.7≤M≤8.1 several equations are very close to the expected size (the × symbol in Figure 4), however, Equations (8)–(14) overestimate the aftershock area for magnitudes M>8.1 while underestimating it for magnitudes below 6. On the other hand, Equation (16) (the □ symbol in Figure 4) follows with a 1% difference in the values of the expected size. We want to emphasize that this equation was derived using the data obtained from the spring-block model, which can be said is the equation governing the synthetic size, and that is why it follows almost perfectly the tendency of the expected size. 

In Table 1 and Table 2, we notice that as the magnitude increases, the difference between the computed and the expected value of the aftershock area also increases. In Table 1, the difference in percentage ranges from 15.48% (for M=7.5) to 270.25% (for M=9.5). Therefore, a better adjustment is needed. For Equation (16), this error ranges from 0.21% (for M=7.5) to 0.26% (for M=9.5). On the other hand, in Table 2, the error for the best fit ranges from 8.4% (for M=6.8) to 297% (for M=9.6), while for Equation (16) the error ranges from 0.16% (for M=6.8) to 0.26% (for M=9.6).

### 3.2. Aftershocks and Utsu’s Law in a Spring-Block Model

**Figure 5:**
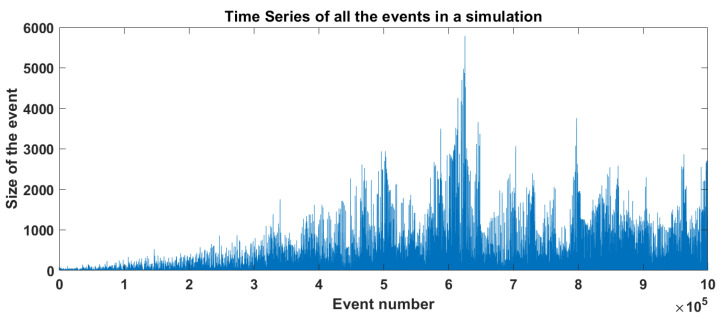
Time series of synthetic earthquakes in the OFC spring-block model.

In Section 2.3, we mentioned the difficulties in accurately reproducing aftershocks in the OFC model and some attempts to statistically identify events as aftershocks. We used a methodology similar to the one used by HN [10] to determine if an event is an aftershock or if it is an isolated event. They used a lattice of 512×512 and $10^{9}$ events. Then, they defined the minimum value for an event to be considered as a mainshock in the time of the mainshock $t_{M}$, which was 1000 blocks, and it had to be the greatest event in an interval of [$t_{M}-10^{-4},{t_{M}+10}^{-4}$]. The next step was to monitor the number of events in a fixed window and subtract the background seismic activity. We used a 100 × 100 model with one million iterations. In Figure 5, we show the time series for the model, the x axis represents the consecutive event number, and since the time between events is always the same (one iteration), the number of events can be considered also as the timestamp. The y axis represents the synthetic size of that earthquake and expresses the total number of relaxed blocks for that iteration. It is worth mentioning that for the first 400 thousand events, they seem to follow an exponential growth before reaching the SOC state. To make the analysis, we had to determine how big an event must be to be classified as a mainshock, we decided to choose only the events whose sizes were 3000 and above that represent a magnitude of 7.2 which is just below the lowest value of the maximum earthquake reported for the subduction regions. Then we chose one of those events and tried to use a window of 10^4^ elements. However, this window was proven to be inefficient since in many cases we found another mainshock inside those 10^4^ elements. So, we decided to use instead of a fixed window just the space between consecutive mainshocks (events with at least 3000 blocks) and tried to elucidate how many of those events could be catalogued as aftershocks. 

**Figure 6:**
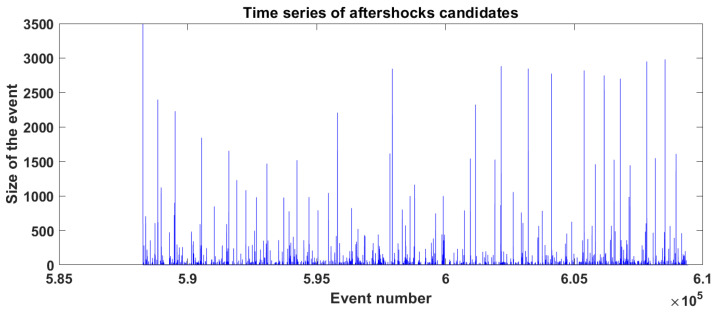
Time series of aftershocks candidates.

**Figure 7:**
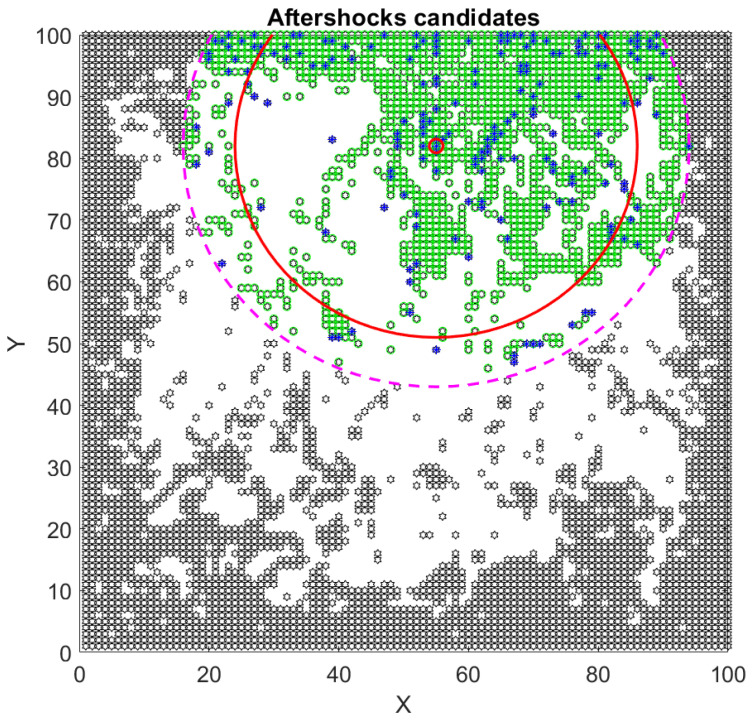
XY plot of all the events between two mainshocks. The dash line represents the initial chosen area while solid line is the aftershock area calculated with Equation (16). Grey hexagons represent all the events that lie outside the rings; green diamonds represent the background noise; blue stars represent the aftershocks candidates.

One crucial difference between our method and the HN one [10] is that they count all the events inside the window without taking into consideration how far they are from the mainshock’s epicenter, while we wanted to relate the event number and its spatial position. For this purpose, we set an arbitrary radius of 40 blocks to delimit the area where to find aftershocks. In Figure 6, we show the time series of the aftershocks’ candidates inside this ring. Then we plot all of these events in an x,y plane as shown in Figure 7. Notice that the events are spread throughout the entire lattice, so it is obvious that not all of them can be considered aftershocks. As mentioned before, we decided to set a radius of 40 (dashed line in Figure 7) choosing the mainshock’s epicenter as the circle’s center, as in many cases presented by Utsu [2]. Once we had this delimitation, we had to eliminate all the events that can be considered as background noise. We set this limit to 27, which is equivalent to events of magnitude M=3.

Then we decided to apply Equation (16) to refine the aftershock area. This new area is shown in Figure 7 as a solid line circle. The mainshock is marked with a small red circle. Events in black hexagons are discarded as aftershocks since they are too far away from the mainshock’s epicenter. The dashed line represents the original area arbitrarily chosen, while the solid line represents Utsu’s aftershock area obtained using Equation (16). Green diamonds were discarded as they represent background noise. Blue stars are the aftershocks candidates.

It is important to mention that many of the biggest events in the interval analyzed were located far away from the mainshock’s epicenter and therefore were discarded as aftershocks.

**Figure 8:**
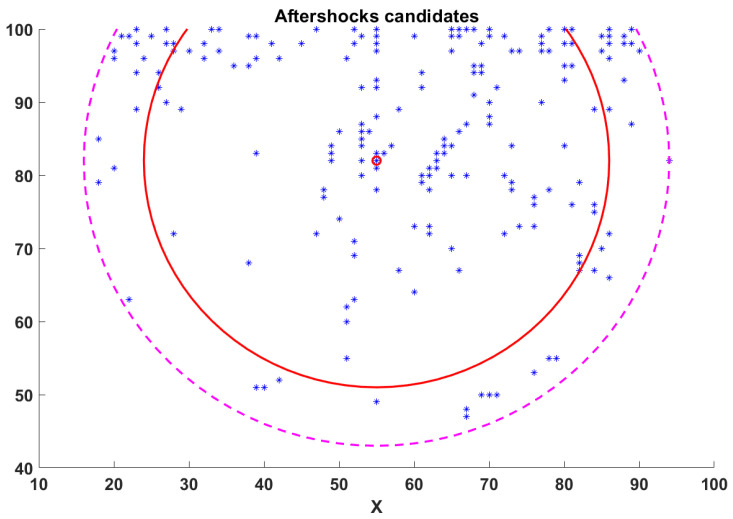
Aftershocks candidates after removing background noise and far away events.

**Figure 9:**
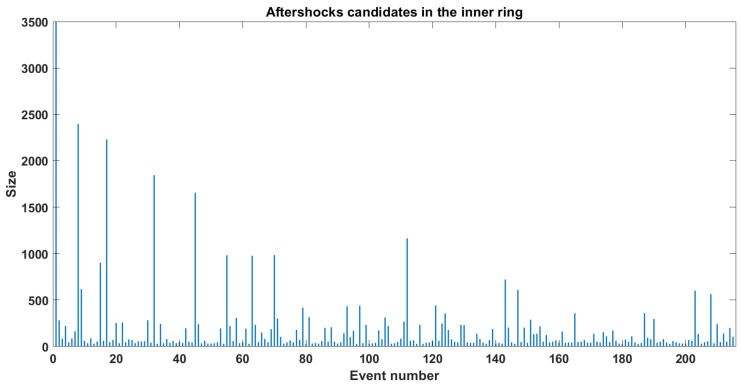
Aftershocks candidates’ series after removing far away events and background noise.

In Figure 8, we present an image of the aftershocks candidates after cleaning the background noise and removing the elements outside the rings. It can be seen that even though we removed several events, we still have enough events enclosed within the rings. What is more, the inner ring, which corresponds to the aftershock area computed with Equation (16), has 216 possible aftershocks which seem reasonable for an earthquake of magnitude M~7. Finally, in Figure 9, we show the time series of those events.

## 4. Discussion

We generated 29 simulations using the OFC model with a 100 × 100 lattice and one million iterations, as in references [5,9]. We applied Equations (8)–(14) for the aftershock area proposed by Utsu and Seki [30], Utsu [2], Goto [31], Bath and Buda [32], and Purcaru [33] to both catalogs: synthetic and real subduction zones as reported in [21]. Finally, we used the data generated by the spring-block model to derive our own Equation (16).

From Table 1 and Table 2, and Figure 4, we observed that when using Equations (8)–(14) the error increases along with the magnitude in both scenarios: real and synthetic. This situation makes it clear that a new fit is necessary. This led us to the proposal of Equation (16). The errors related to this equation are on the order of 0.2%. It is clear that for both cases, the best equation to approximate the size of the event is Equation (16). This might stand, therefore, as an indicator that validates this proposal. Although this result was obtained using the spring-block model, its application to real events (complete data sets) is yet to be analyzed. 

**Figure 10:**
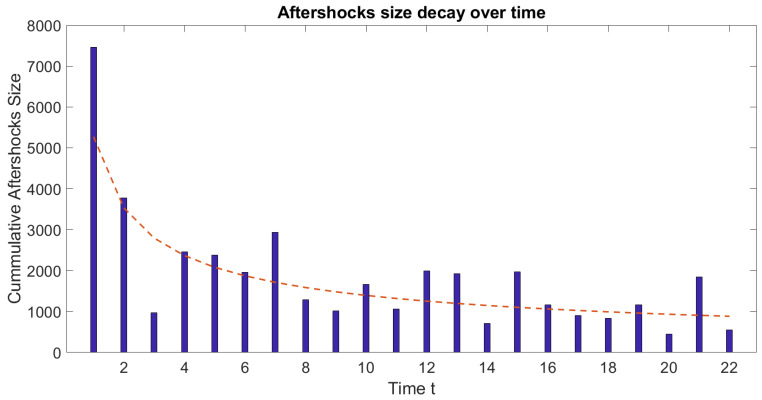
Aftershocks decay with time. The dotted red line represents the adjusted decay over time that follows $y=5270.2t^{-0.578}$.

Regarding the identification of aftershocks candidates in the spring-block model, we believe that it is possible that we can classify some events as aftershocks, especially if we use, as a discriminant analysis, the spatial location and not only the recurrence time. However, we admit that there is a lot of work to do on this matter, especially since we have not proven that these candidates follow Omori’s law for aftershocks [11]. From Figure 9, one might rush and say that they follow a hyperbolic decay. Data from Figure 9 is kind of reminiscent of Omori’s law, where the cumulative size of the aftershocks decays with time following the equation $y=5270.2t^{-0.578}$ as illustrated in Figure 10. The behavior of the aftershocks is remarkably similar to Omori’s law with the difference that we do not show the decay in the number of events but in their cumulative size (this size as mentioned earlier is the number of relaxed blocks per event). Eventually, they become small enough to be considered as part of the background activity, similar to the real-life seismic activity.

## 5. Conclusions

We conclude that it seems possible to qualitatively reproduce Utsu’s law using the OFC spring-block model due to the similarities between the actual seismic regions compared to the simulations. It is clear that a single equation cannot fit all ranges of magnitudes in real scenarios. For the synthetic case, we propose an Equation (16) that fits the expected values very well. This does not mean that the same behavior can be expected in real seismicity since we used synthetic catalogs to derive that equation, but it does make clear the need for a better spring-block model that qualitatively approaches the real-life seismicity. With this, we are not saying that the current model is not valid. It is important to remember that this is a model that works with quite simple rules and does not include many of the parameters observed in real seismicity. However, it fulfills the function of qualitatively reproducing many of the empirical laws and behaviors found in the real world, and for this study, it provides a good approximation for certain quantities where all other equations seem to work well. 

Finally, although our results seem to confirm that Utsu’s law can be reproduced in the spring-block model, a deeper analysis of the possible replications in the OFC model is necessary. In addition, the inclusion of spatial location when identifying possible aftershocks brings us closer to a more complete model of earthquakes. It should be noted that these results are promising regarding the use of the spring-block model and its ability to reproduce complex properties of the seismic phenomenon.

## Figures and Tables

**Table 1:** Comparison of the aftershock area given by Equations (8)–(14) using the maximum real earthquake’s magnitudes reported in each seismic region. The expected size was computed by using Equation (15). The closest value to the expected size is highlighted in green. The last column is the result of our proposed Equation (16), which gives the best approximation to the expected size.

Subduction Seismic Region	M	Expected Size	Equation (8)	Equation (9)	Equation (10)	Equation (11)	Equation (12)	Equation (13)	Equation (14)	Equation (16)
Caribbean	7.5	3788	4365	6310	2512	1349	1259	10,593	8511	3780
Marianas	7.5	3788	4365	6310	2512	1349	1259	10,593	8511	3780
Scotia arc	7.6	4228	5521	7943	3162	2014	1585	13,996	10,914	4219
S. Java	7.7	4719	6982	10,000	3981	3006	1995	18,493	13,996	4709
New Zealand	7.8	5267	8831	12,589	5012	4487	2512	24,434	17,947	5255
Patagonia	7.8	5267	8831	12,589	5012	4487	2512	24,434	17,947	5255
Vanatu (New Hebrides)	7.9	5878	11,169	15,849	6310	6699	3162	32,285	23,014	5865
Izu Bonin	7.9	5878	11,169	15849	6310	6699	3162	32,285	23,014	5865
Kermadec	7.9	5878	11,169	15,849	6310	6699	3162	32,285	23,014	5865
México-Oaxaca	7.9	5878	11,169	15,849	6310	6699	3162	32,285	23,014	5865
México-Guerrero	7.9	5878	11,169	15,849	6310	6699	3162	32,285	23,014	5865
Ryukyus	8	6,561	14,125	19,953	7943	10,000	3981	42,658	29,512	6546
Tonga	8.1	7323	17,865	25,119	10,000	14,928	5012	56,364	37,844	7306
Peru	8.1	7323	17,865	25,119	10,000	14,928	5012	56,364	37,844	7306
Central America	8.1	7323	17,865	25,119	10,000	14,928	5012	56,364	37,844	7306
Mexico-Michoacán	8.1	7323	17,865	25,119	10,000	14,928	5012	56,364	37,844	7306
W. Alaska	8.2	8173	22,594	31,623	12,589	22,284	6310	74,473	48,529	8155
México- Jalisco	8.2	8173	22,594	31,623	12,589	22,284	6310	74,473	48,529	8155
Sumba Island	8.3	9122	28,576	39,811	15,849	33,266	7943	98,401	62,230	9101
Kuriles	8.5	11,364	45,709	63,096	25,119	74,131	12,589	171,791	102,329	11,337
West Aleutians	8.6	12,684	57,810	79,433	31,623	110,662	15,849	226,986	131,220	12,653
Komandorski	8.7	14,156	73,114	100,000	39,811	165,196	19,953	299,916	168,267	14,122
Central Chile	8.8	15,800	92,470	125,893	50,119	246,604	25,119	396,278	215,774	15,762
Colombia-Ecuador	8.8	15,800	92,470	125,893	50,119	246,604	25,119	396,278	215,774	15,762
Kamchatka	9	19,683	147,911	199,526	79,433	549,541	39,811	691,831	354,813	19,634
NE Japan	9.1	21,969	187,068	251,189	100,000	820,352	50,119	914,113	454,988	21,913
Sumatra-Andaman	9.2	24,520	236,592	316,228	125,893	1,224,616	63,096	1,207,814	583,445	24,457
East Alaska	9.2	24,520	236,592	316,228	125,893	1,224,616	63,096	1,207,814	583,445	24,457
South Chile	9.5	34,092	478,630	630,957	251,189	4,073,803	125,893	2,786,121	1,230,269	34,002

**Table 2:** Comparison of the aftershock area given by Equations (8)–(14) using the spring-block model. For each simulation, we obtained the maximum synthetic size of a given region, then the magnitude M was computed applying Equation (15) and solving for $M=\log_{3}\hat{S}$. The closest value to the expected size is highlighted in green. The last column is the result of our proposed Equation (16), which gives the best approximation to the synthetic size.

Subduction Seismic Region	M	Synthetic Size	Equation (8)	Equation (9)	Equation (10)	Equation (11)	Equation (12)	Equation (13)	Equation (14)	Equation (16)
Caribbean	6.8	1832	924	1376	548	95	275	1678	1644	1829
Marianas	7.2	2738	2181	3195	1272	413	638	4650	4082	2732
Scotia arc	7.2	2786	2263	3314	1319	440	661	4860	4246	2780
Vanatu (New Hebrides)	7.6	4187	5407	7783	3099	1944	1553	13,655	10,677	4178
Izu Bonin	7.6	4216	5488	7897	3144	1993	1576	13,896	10,845	4207
S. Java	7.7	4561	6493	9312	3707	2655	1858	16,964	12,959	4551
Kermadec	7.7	4598	6606	9471	3770	2735	1890	17,315	13,198	4588
New Zealand	7.7	4610	6643	9523	3791	2761	1900	17,430	13,276	4600
Sumba Island	7.8	5175	8505	12,134	4831	4209	2421	23,369	17,247	5164
Patagonia	8.0	6298	12,942	18,313	7291	8614	3654	38,454	26,902	6284
Tonga	8.1	7099	16,718	23,536	9370	13,330	4696	52,096	35,276	7083
NE Japan	8.2	7959	21,347	29,910	11,908	20,227	5968	69,622	45,697	7941
Kuriles	8.2	8405	23,986	33,531	13,349	24,677	6690	79,946	51,700	8386
Ryukyus	8.3	9043	28,047	39,088	15,561	32,223	7799	96,245	61,012	9022
Kamchatka	8	9994	34,732	48,202	19,190	46,402	9618	124,025	76,509	9971
W. Alaska	8.4	10,054	35,179	48,811	19,432	47,426	9739	125,923	77,553	10,030
Komandorski	8.4	10,124	35,705	49,526	19,717	48,641	9882	128,158	78,780	10,100
Sumatra-Andaman	8.4	10,283	36,914	51,170	20,371	51,486	10,210	133,324	81,609	10,259
West Aleutians	8.4	10,352	37,446	51,892	20,659	52,757	10,354	135,604	82,853	10,328
Peru	8.4	10,572	39,168	54,231	21,590	56,962	10,820	143,033	86,893	10,547
East Alaska	8.6	13,084	61,781	84,779	33,751	123,944	16,916	245,601	140,784	13,053
Central America	8.7	14,301	74,719	102,152	40,667	171,431	20,382	307,743	172,181	14,266
Central Chile	8.8	15,020	82,980	113,213	45,071	205,012	22,589	348,509	192,401	14,983
México-Oaxaca	8.9	18,605	131,131	177,309	70,588	447,499	35,378	599,742	312,341	18,559
México-Guerrero	9.1	21,842	184,770	248,163	98,795	803,235	49,515	900,807	449,072	21,787
Mexico-Michoacán	9.3	26,583	281,198	374,578	149,122	1,644,232	74,738	1,482,470	700,528	26,514
Colombia-Ecuador	9	28,450	325,111	431,843	171,920	2,106,040	86,164	1,760,938	816,868	28,376
México-Jalisco	9.4	29,780	358,469	475,240	189,197	2,487,879	94,823	1,977,267	905,871	29,702
South Chile	9.6	36,428	551,485	724,982	288,621	5,187,635	144,653	3,296,066	1,429,399	36,331

## Data Availability

Synthetic data can be provided by writing to the corresponding author.
